# Supplementary material for: Are carbon emissions trading and green financial instruments synergistic? -Comprehensive quantitative research based on content analysis
Source: PLoS One. 2024 Mar 7;19(3):e0298601. doi: 10.1371/journal.pone.0298601 (PMC10919601; doi:10.1371/journal.pone.0298601)
Supplement: S1 Data — (ZIP) [file pone.0298601.s001.zip › Data/地级市Z_F工作报告.zip!/╕╩╦α/╛╞╚¬╩╨/└┤╫╘╣1⁄2╓┌║┼í╛┬φ┐╦╩2╛▌═°í┐_2018.docx]

各位代表:

现在，我代表市人民政府向大会作工作报告，请予审议，并请各位政协委员和列席人员提出意见。

2017年工作回顾

过去的一年，面对经济形势复杂多变、下行压力持续加大、多种困难层层叠加的严峻挑战，在省委、省政府和市委的坚强领导下，我们认真学习贯彻十八大以来各项重大决策部署特别是党的十九大精神，以习近平新时代中国特色社会主义思想为指导，全面落实习近平总书记视察甘肃重要讲话和“八个着力”重要指示精神，按照省第十三次党代会和市第四次党代会工作部署，深入推进供给侧结构性改革，聚力攻坚克难，积极主动作为，较好完成了四届人大一次会议确定的各项任务。

——经济运行质量提高。预计，全市完成生产总值594亿元，农业生产和消费需求平稳，石化、电力、医药等重点工业行业较快增长；规上工业主营业务利润率提高7.1个百分点，实现利税增长77.5%；财政总收入110.3亿元，增长7.6%。

——“三去一降一补”成效明显。煤炭、水泥产能压减任务完成，规上工业产成品库存下降12.1%，商品住宅去化周期缩减到合理范围。置换优化政府存量债务11.5亿元，工业企业资产负债率同比下降1.2个百分点。取消行政事业性收费22项，企业职工“五险”费率调降4.3个百分点，促使规上工业企业节本增效28亿元。

——动能转换加速推进。研发投入实现增加值4.1亿元，增长7.5%。省级以上众创孵化平台达到12个。全年注册登记企业3559户，加上个体工商户和农民专业合作社，平均每天新增各类市场主体40.2户，培育增加规上民营企业20户，非公经济比重提高到53.6%。

——脱贫攻坚步伐加快。移民人均可支配收入达到9200元，增长15%，高于全市农民人均收入增速7.5个百分点，37个贫困村有33个达到脱贫退出标准，预脱贫人口1.4万人，贫困发生率下降到0.44%，玉门市、瓜州县达到省上脱贫摘帽验收标准。

——生态建设力度加大。压茬推进各类自然保护区项目排查整改，依法关停探、采矿权303个。新增人工造林20万亩、封滩育林10万亩，完成义务植树404万株。城镇建成区绿化覆盖率达到38.7%，人均公共绿地面积达到13.6平方米。万元GDP能耗和工业增加值用水量控制在省定目标。

——民生福祉持续改善。新增就业岗位2.6万个，城镇登记失业率控制在2.7%。城镇居民人均可支配收入32478元，农村居民人均可支配收入15690元，分别增长8%和7.5%。政府承诺的高校毕业生见习就业、职业技能培训、城乡残疾老人困难老人生活补助提标等10件惠民实事全部落实，改革发展成果更多更公平地惠及人民群众。

这些成绩的取得，标志着我们在建设活力小康生态魅力幸福酒泉的征程上迈出了坚实的一步。一年来所做的主要工作是:

1.加快推进改革创新，活力酒泉建设有了新进展

重点领域改革全面深化。着眼增强供给侧结构性改革的协同性，出台了农业水权水价、政府购买服务等一系列改革方案和实施意见，各项改革呈现出纵深推进、重点突破的良好态势。扎实开展“放管服”改革，深化负面清单制度，行政审批事项削减41%。农村“三权”分置改革、农业设施产权登记、农业水权水价改革全面推开，农村土地承包经营确权登记颁证工作基本完成。深入推进商事制度改革，实施“多证合一”工商登记，群众办事更加方便快捷。深化财政改革，加快融资平台市场化转型，戈壁农业产业发展基金完成工商注册，浙商银行酒泉分行批准筹建，小额贷款公司监管加强，金融风险得到有效管控。

科技创新引领作用明显。深入贯彻国家创新驱动发展战略，出台支持科技创新促进科技成果转化工作方案，投入科技扶持引导资金4410万元，实施科技项目118项，建成省级以上工程技术研究中心(工程实验室)18个，高新技术企业达到37户，新培育创新型企业58户，科技进步贡献率达到56%。

项目带动战略深入实施。把扩大有效投资作为应对经济下行的重要举措，推行重大项目联审联批和“一站式”服务，完善领导包挂、部门包抓机制，一批调结构、补短板、增后劲的重大项目落地建设。酒泉核技术产业园加快建设，瓜州县新能源供暖等民生项目建成，敦煌首航、玉门鑫能等光热发电示范项目开工建设。加大招商引资力度，引进到位资金549亿元。

产业结构调整积极推进。围绕农业供给侧结构性改革，大力发展戈壁农业，新增日光温室5260亩、钢架蔬菜大棚1.9万亩，定植特色林果10.6万亩，建成戈壁农业产业园39个，蔬菜、林果、制种、中药材、牧草业等五大特色产业更加鲜明。坚持转型与升级并重推进新型工业化，认真落实工业“止滑稳增”和“三重”“三一”政策措施，对停减产企业实行“一企一策”包挂帮扶，引入战略投资重组复产三新硅业，停减产企业复产率稳步提升；酒湖正负800千伏特高压直流输电工程并网运行，弃风弃光问题逐步缓解；积极推进售电侧改革试点，落实电力直购优惠政策，肃州、玉门微电网和瓜州、玉门风电平价上网示范项目获批。着力提升三产发展水平，酒泉万达广场、瓜州红星美凯龙家具博览中心建成开业，社会消费品零售总额增长8%。加快文化旅游融合发展，文化演艺、体育健身等服务业态快速增长，旅游接待人数和旅游收入分别增长23.6%和28.7%。

2.统筹城乡全面发展，小康酒泉建设迈出新步伐

脱贫攻坚扎实推进。强化领导包抓、部门帮扶工作机制，聚焦建档立卡贫困村、贫困人口，靠实帮扶责任，整合投入扶贫资金4.2亿元，改善移民乡村基础设施，改良土地3万亩，衬砌渠道210公里，新建乡村道路140公里，新建村级光伏电站27座。积极培育富民产业，新增枸杞、设施果蔬2.6万亩，特色高效作物种植面积达到20万亩，肉羊饲养量达到40万只。精准落实教育扶贫、健康扶贫、兜底保障等政策，脱贫攻坚质量显著提升。

城市基础设施全面提升。《酒泉市城市总体规划(2016—2030年)》和玉门、敦煌“多规合一”批复实施，我市被确定为丝绸之路经济带甘肃段重要节点城市和省域副中心城市。酒泉城区新建改建光电大道、酒银路、公园路等18条道路，热电联供一级管网优化改造，两家燃气公司整合重组。肃州湿地景观大道、金塔航天大道、玉门风光大道、瓜州榆林大道建成通车，肃州四坝海子湿地公园、敦煌飞天公园、肃北紫亭湖千亩花田、阿克塞民族新村建设各具特色，全市棚户区改造年度任务超额完成。

城乡一体化加快推进。深入推进肃州区中小城市综合改革试点和敦煌市新型城镇化综合改革试点，出台特色小镇建设实施意见。加强水利、交通设施建设，农村安全饮水提升工程惠及5.2万人，党河灌区信息管理系统、敦煌城市地下水源地置换等项目开工建设，瓜敦高速、玉昌公路等重点交通项目建成通车，肃航一级公路建设进展顺利，新改建农村公路1282公里。改造农村危旧房733户，建成美丽乡村示范村36个。全面实施居住证制度，引导农业转移人口进城落户，城镇化率提高1.6个百分点。

3.坚持绿色发展理念，生态酒泉建设落实新要求

生态安全屏障积极构筑。坚守生态环保底线，落实草原、森林奖补政策，加大“三北防护林”、生态公益林和重点风沙口治理，金塔防沙治沙和瓜州文化创新区生态治理取得明显成效。加强沙化土地封禁保护修复，各级财政投入防沙治沙资金2.9亿元，治理沙化土地45.2万亩，有效减少了风沙侵害。肃州花城湖、金塔北海子等湿地保护与恢复项目组织实施，敦煌水资源合理利用与生态保护项目有序推进。

生态环境保护力度加大。汲取祁连山生态环保问题的深刻教训，对自然保护区及其他生态敏感区内项目清理排查，各类违建项目和生产活动依法关停并正在有序退出，中央、省上环保督察交办的信访案件全面整改到位，实现“河长制”全覆盖。严格落实国家大气、水、土壤污染防治行动计划，实施了一批减排治污项目，强化工业排放和建筑工地扬尘管控，加大燃煤锅炉改造关停、黄标车老旧车淘汰和露天烧烤摊点取缔力度，四项污染物减排任务全面完成，市区空气优良天数达到301天，比上年增加56天。

城乡环境整治扎实有效。全民动员开展城乡环境综合整治突破行动和全域无垃圾专项治理行动，集中整治道路沿线、旅游景区、农村集镇、村庄院落、城乡接合部等区域“脏乱差”，一批农宅风貌改造、道路硬化亮化、垃圾污水处理、保洁清运设施等项目建成，部分乡镇和园区的垃圾污水集中处理设施开工建设，农村基础条件和人居环境明显改善。深入推进爱国卫生运动，定期清扫、垃圾清运、门前“三包”、公共设施管护等长效机制普遍建立，城乡环境整治取得阶段性成效。

4.着力扩大对外开放，魅力酒泉建设展现新面貌

文博效应逐步显现。全力推进场馆完善、环境营造、会务保障等工作，成功举办第二届丝绸之路(敦煌)国际文化博览会，高峰论坛、文化年展、文艺演出等主题活动丰富多彩。拓展承办“一带一路”媒体合作论坛和司法合作国际论坛，提升了文博会层次，扩大了对外影响力。敦煌国家级文化产业示范园区建设加快，带动旅游业持续升温，《又见敦煌》《敦煌盛典》持续火爆，《丝路花雨》实现常态化驻场演出，全年开展各类文化演艺活动1100余场次。

对外开放步伐加快。积极融入“一带一路”建设，力促新能源、装备制造、种子产业开展国际产能合作，扩大脱水蔬菜、番茄制品等农产品出口基地建设，光伏逆变器、温性石棉出口实现零的突破。协调开通乌鲁木齐至敦煌旅游专列，新增敦煌至鄂尔多斯、杭州、深圳等城市航线和香港、大阪至敦煌旅游包机。积极搭乘“中新南向通道”，推进陆海贸易新通道物流节点建设，石棉等矿产品纳入首发班列出口货源。全市引进外资1326万美元，实现进出口总额4.8亿元。

合作交流领域拓宽。聚力打造户外运动之都，举办第三届酒泉国际戈壁超级马拉松、第十二届“玄奘之路”商学院戈壁挑战赛等户外活动，肃州区、玉门市被确定为河西走廊国家体育产业示范基地。主动“走出去”招商引资，在重庆、杭州等城市举办经贸洽谈活动，组织企业参加广交会、哈萨克斯坦(中国)商品展览会、美国食品配料展等国内外展会，与上海江桥、广西桂林分别签署优质农产品销售和文化旅游发展合作协议。

5.切实保障改善民生，幸福酒泉建设取得新成效

社会保障加快完善。各级财政投入养老、社保、就业等民生资金43.14亿元，占一般公共预算支出的比重达31%。多渠道开发就业岗位，落实“双创”扶持政策，输转农村劳动力20万人次，安置高校毕业生、就业困难人员、零就业家庭就业4331人。全面推行城镇职工“五险合一”统筹经办新模式，城乡医疗保险并轨运行，企业退休人员养老金、城乡低保等保障标准大幅提高，城乡居民养老保险续保率达96%、医疗保险参保率保持在98%以上。

社会事业全面发展。落实教育惠民资助政策，学前教育保教费补助、贫困家庭学生救助等补助资金足额发放，新建维修校舍19.1万平方米，酒泉第七中学主体封顶，玉门市第三小学和第四幼儿园建成招生，城镇义务教育大班额得到缓解。公立医院全面取消药品加成，医院人事薪酬制度改革和医联体建设启动实施，跨省就医实现联网异地结算。加强公共文化服务，一批基层文化中心和“乡村舞台”建成投用，文化节会活动精彩纷呈。民族地区项目帮扶深入推进，民族团结、宗教和顺局面进一步巩固。统计调查、气象服务、人防建设继续加强，妇女儿童、老年人和残疾人权益得到有效保障，档案、史志、侨务、双拥等工作有序推进。

平安酒泉建设扎实推进。落实信访工作责任制，一批信访积案和上访问题得到解决。深入推进“七五”普法，持续开展“打非治违”和安全隐患排查整治，全年没有发生重特大安全生产事故。加强防震减灾救灾能力建设和突发公共事件应急管理。大力开展食品药品专项整治，食品药品安全形势持续向好。完善社会治安防控体系，严厉打击非法集资、电信诈骗、金融诈骗等违法犯罪，人民群众的安全感、满意度明显提升。

各位代表，一年来，我们牢固树立“四个意识”，严格执行中央八项规定精神和省、市委相关规定，坚决全面肃清王三运、马光明流毒和影响，深入开展“明查暗访督查年”活动和“三纠三促”专项行动，促进了党风政风和干部作风明显好转。自觉接受人大法律监督、工作监督和政协民主监督，办理人大代表建议153件、政协委员提案211件。严格财政预算管理，“三公”经费支出下降25.6%。加大审计监督力度，查处违规和管理不规范资金近5亿元。认真履行“一岗双责”，严肃查处违规违纪案件，纠正了一些部门和单位推诿扯皮、效率低下、损害群众利益的不正之风。

各位代表，2017年我们经受住多重困难和压力挑战，取得了来之不易的成绩。这是省委、省政府和市委坚强领导的结果，是市人大、市政协和社会各界大力支持、有效监督的结果，是全市人民辛勤努力、共同奋斗的结果。在此，我代表市政府，向各位人大代表、政协委员和全市广大干部群众，向各民主党派、工商联、人民团体、离退休老同志，向驻酒人民解放军、武警官兵和公安干警，向中央、省属驻酒单位以及所有关心、支持酒泉发展的各界人士表示衷心的感谢!

在肯定成绩的同时，我们也清醒地认识到，当前的发展还面临许多困难和问题。一是经济下行压力依然较大，投资消费支撑减弱，生产总值出现负增长，工业生产降幅较大，服务业增长乏力，止滑稳增任务艰巨。二是受国家对风电项目限批、生态环保安全政策更加严格、重大项目储备不足、招商项目落地难等因素影响，固定资产投资下降50.6%。三是现代农业体系不健全，土地流转规模小，农民组织化程度低，农产品精深加工转化水平不高，农民持续增收的难度较大。四是财政收支矛盾突出，财源结构不合理，非税收入占比高，财政保障和债务化解压力较大。五是各级政府破解发展难题的能力不足、措施不多、效果不明显，个别部门主动服务意识不强，一些干部不担当、不作为问题突出，抓落实力度不够，影响了政府工作效能。对于这些问题，我们将认真研究，切实采取措施，努力加以解决。

2018年主要目标和重点任务

2018年，是贯彻党的十九大精神的开局之年，是改革开放40周年，也是决胜全面建成小康社会、实施“十三五”规划承上启下的关键一年。党的十九大作出了中国特色社会主义进入新时代和我国社会主要矛盾发生变化的重大判断，经济由高速增长阶段转向高质量发展阶段，这为我们决胜全面建成小康社会提供了根本遵循。中央经济工作会议和省委、省政府确立的打好三大攻坚战、建设现代化经济体系等重点任务，为我们破解发展瓶颈、推动产业转型升级指明了方向。酒泉处在全省经济社会发展“第一方阵”，但近几年位次下移，支撑全省发展的贡献减弱，与省域副中心城市定位不相称，与省委、省政府对酒泉发展的高度重视和人民群众的热切期望还有差距。我们必须紧跟新时代步伐，顺应新时代要求，把人民对美好生活的向往作为奋斗目标，坚定不移把发展作为第一要务，抓住“一带一路”建设这一最大机遇，变被动为主动、变压力为动力，坚定信心，攻坚克难，打好产业转型升级仗，不断开创高质量发展新局面。

2018年政府工作的总体要求是:坚持以习近平新时代中国特色社会主义思想为指导，全面贯彻党的十九大精神，深入贯彻习近平总书记视察甘肃重要讲话和“八个着力”重要指示精神，认真落实省第十三次党代会和市第四次党代会决策部署，坚持稳中求进工作总基调，坚持新发展理念，按照高质量发展的要求，统筹推进“五位一体”总体布局，协调推进“四个全面”战略布局，坚持以供给侧结构性改革为主线，全力做好稳增长、促改革、调结构、惠民生、防风险各项工作，全面深化改革创新，大力推进乡村振兴，加快培育现代产业，持续扩大有效投资，积极保障改善民生，奋力推进活力酒泉、小康酒泉、生态酒泉、魅力酒泉、幸福酒泉建设。

2018年的主要预期目标是:全市生产总值增长5%左右，固定资产投资增长7%，社会消费品零售总额增长8%，一般公共预算收入增长5%，城镇居民人均可支配收入增长7%，农村居民人均可支配收入增长8%，单位生产总值能耗下降2%，居民消费价格指数涨幅控制在2.5%以内。在实际工作中，各项指标争取更好结果。

为实现上述目标，我们将重点抓好九个方面的工作:

1.全面深化改革创新，激发市场主体活力

把改革创新贯穿于经济社会发展各个领域，坚持问题导向，紧盯重点难点，推进基础性关键性领域改革取得新突破，着力增强经济内生动力。

加快供给侧结构性改革。着眼推动经济发展质量变革、效率变革、动力变革，深化要素市场化配置改革，健全资源市场化配置机制，建立完善环保税、资源税征缴制度，严格落实环保、能耗、用地、质量、安全标准，形成激励先进企业兼并重组、倒逼“僵尸企业”退出的政策导向。认真落实降低企业成本实施方案，清理规范政府性基金和涉企收费，切实降低企业用电、用工、用地、物流等成本。强化金融支持实体经济的能力，防范化解金融风险，推进政府融资平台市场化转型，戈壁农业产业发展基金完成投放，支持企业通过债转股、股权债权融资等方式“去杠杆”。发展绿色金融，推进建立有利于节能环保、清洁生产等绿色产业发展的体制机制。严格控制政府债务增量，加大存量债务置换力度，规范PPP项目及政府购买服务，确保把政府债务率控制在合理水平以内。进一步整治小额贷款公司借贷行为，坚决取缔“地下钱庄”，严厉打击套取贷款、非法变相吸收公众存款、非法集资、高利贷等违法违规金融活动，打好防范化解重大金融风险攻坚战。

推动农村“三变”改革。加快农村资源变资产、资金变股金、农民变股东改革，在肃州区、玉门市先行试点。完善农村产权制度和要素市场化配置，开展农村集体“三资”清产核资，界定农村集体经济组织成员资格，有序开展确权登记，引导农民以土地承包经营权和林权入股，积极推进集体资产入股，规范精准量化股权，推进土地承包经营权流转，提高规模化经营水平。引进培育龙头企业、专业合作社、家庭农场等新型农业经营主体，引导农民入股经营主体，开展多种形式的农村股份合作经营。

深化“放管服”改革。继续推进简政放权，整合取消关联、相近类别审批事项，着力提高承接和服务能力。建立行政审批事项“预下放”制度，推动审批链同步下放，增强协同性、联动性。强化事中事后监管，落实“双随机一公开”制度。改进政务服务，优化行政审批，促进政府部门放管结合、优化服务。深化“多证合一”“证照分离”商事制度改革，放宽市场主体经营场所登记条件。落实投资项目并联审批制度，促进线上线下审批无缝衔接，全面推行审批承诺制和代办制，进一步优化营商环境。

2.实施乡村振兴战略，发展壮大县域经济

把推动农业全面升级、农村全面进步、农民全面发展作为新时代乡村振兴的重要抓手，加快农业农村现代化，促进城乡产业融合，让广大城乡群众过上更加幸福美好的新生活。

大力发展戈壁农业。坚持质量兴农、绿色兴农，认真落实戈壁农业发展政策措施。按照规划一步到位、建设分步实施的思路，主攻戈壁温室、连片大棚，新建高标准设施农业5万亩以上，新植特色林果5万亩，新增肉羊出栏量50万只，推广农田节水技术182万亩。建立温室建造、有机生产、质量检测标准体系，打造“戈壁雪润”有机农产品品牌，确保农产品质量安全。提高农民组织化程度和农业产业化经营水平，扶持发展一批种养殖专业合作社，壮大农产品储藏运销和精深加工龙头，积极开拓农产品外销市场，引导农业发展由“生产导向”向“消费导向”转变。

打好精准脱贫攻坚战。以玉门、瓜州移民乡村和贫困人口为重点，打好基础设施、产业扶贫、政策兜底“组合拳”，改善贫困乡村生产生活条件。学习借鉴“庄浪模式”，扎实推进产业脱贫，用足用好产业扶贫基金和扶贫贷款延期政策，依托枸杞、蔬菜、中药材、设施养殖等特色优势产业，引进培育农业经营主体，做强农民专业合作社，推动农户与专业合作社、龙头企业建立紧密的利益共同体，让贫困农户获得更多更稳定的财产性收入。着力解决贫困村集体经济薄弱的问题，新建50千瓦村级光伏电站24个。落实低保兜底政策，关注因病返贫、因灾返贫问题，实现应扶尽扶、应保尽保，确保建档立卡贫困人口收入增幅高于全市平均水平，移民人均可支配收入增长10%。

加快城乡融合发展。按照产业兴旺、生态宜居、乡风文明、治理有效、生活富裕的总要求，健全城乡融合发展机制，加快形成工农互促、城乡互补、全面融合、共同繁荣的新型工农城乡关系，促进城乡一体、城乡和谐。持续改善农村人居环境，加大重点集镇和偏远乡村基础设施建设投入。加快美丽乡村、特色小镇、休闲农庄和旅游示范村建设，支持发展集农业生产、旅游观光、四季采摘、农耕体验、餐饮住宿为一体的田园综合体，着力打造一二三产融合发展、生产生活生态同步改善的新农村。

培育发展县域经济。坚持因地制宜、错位发展，以县城为中心、乡镇为纽带、农村为腹地，加强规划引导，明确目标定位，强化扶持政策集成，创新区域资源整合，构建“一县一特、一乡一业、一村一品”的县域经济格局，促进县域经济特色化、差异化发展。围绕特色产业发展，优化提升现代农业示范区、经济技术开发区、工业集中区和各类农业科技园、高新技术产业园、文化旅游产业园等特色园区，促进市场要素优化配置，推动产业集约集群发展，着力构建“一区多园”、产业互补、特色鲜明、功能完善的县域经济增长极。探索引进社会资本参与园区基础设施建设和运营管理，提高项目承接和公共服务水平。

3.推进工业强市战略，构建现代产业新体系

坚持改造传统产业与发展新兴产业并举，推进工业转型发展、集群发展、绿色发展，提升产业发展质量，加快建设实体经济、科技创新、现代金融、人力资源协同发展的产业体系。

持续优化清洁能源产业。围绕创建国家新能源综合示范区，坚持优化存量与提升增量并重、本地消纳与跨区外送并举，调整提升清洁能源产业。认真落实新能源产业与装备制造、先进高载能产业互保共建、联动发展的政策措施，探索装备制造、清洁能源与电能消纳互补发展新模式，积极开拓酒湖直流受端地区电力市场，支持瓜州、玉门开展大用户直供电交易。推进肃州、瓜州、敦煌增量配电改革试点和肃州、玉门微电网示范项目，用好用足价格政策，加快发展清洁能源供暖，促进清洁能源就地消纳。

转型提升传统优势产业。立足资源优势和产业基础，加快石油化工、有色冶金、建筑建材等传统产业绿色化、循环化改造，延伸拓展产业链，推进资源综合利用，推广绿色清洁生产工艺，规模化、集群化、高端化发展石材加工业，促进重化工业和传统产业提质增效。支持玉门油田二次开发，推进油田产能建设和煤岩油气资源综合开发利用。加快洋葱、肉羊、葡萄、枸杞等特色农产品由初级加工向精深加工转变，提升产加销一体化发展水平。

培育壮大战略新兴产业。深入实施《中国制造2025酒泉行动计划》，着力发展智能制造、新型材料、生物医药、节能环保、大数据等新兴产业。加快大禹节水、奥凯种机智能制造生产线建设，促进装备制造向数字化、智能化方向发展。积极引进新型装配式建筑材料，推广应用绿色环保建材。启动建设中以节水产业园，支持西域新材、三新硅业等新兴产业骨干企业发展壮大，加快开发复合新材料等高附加值产品。深化与浪潮集团战略合作，推动建设酒泉云计算中心。

协同推进军民融合产业。建立军民融合发展领导协调机制，加强与中核四○四公司、酒泉卫星发射中心、空军训练试验基地等军工企业和国防科研单位的项目合作，积极搭建军民融合创新平台，促进军民资源互通共享、相互支撑和有效转化。全力保障中核酒泉核产业园建设，主动与核技术研究院所沟通对接，谋划引进核素新材料关联配套项目，做好“核电宝”示范合作项目前期工作，支持玉门北山高放射性物质深层处理实验项目。加快跟进金塔西川园区基础设施建设和配套产业引进，推进核设备制造、低放减容处理等核关联项目。

全力帮助企业脱困增效。认真落实“三重”“三一”实施方案，狠抓停产企业复产、减产企业达产、小微企业升规入库工作。采取领导包挂、驻企帮扶等措施，依法依规帮助企业解决实际困难，促其正常生产经营。深入推进输配电价改革、直购电交易、综合电价扶持和商业峰谷分时电价试点，落实电价优惠政策，切实降低企业用电成本。健全政银企融资对接机制，扩大“助保贷”“互助贷”规模，着力缓解企业融资困难。

4.多元发展第三产业，形成合作开放新格局

积极融入和服务“一带一路”倡议，大力发展现代服务业和外向型经济，构建全方位、多层次的对外开放新格局。

加快发展文化产业。深入实施“十三五”文化产业发展规划，大力推进我市5处世界文化遗产和敦煌文化、航天文化等特色文化资源的发掘与开发，努力把文化资源优势转化为经济发展优势。进一步提升内涵、拓展外延、突出效益、强化保障，精心办好第三届丝绸之路(敦煌)国际文化博览会，为推动“一带一路”倡议、促进国际文化合作和文明交流做出新的贡献，进一步提升酒泉知名度和影响力。全面落实文化旅游产业发展实施意见，深入实施“文化＋”战略，促进文化、旅游、体育融合发展，加快敦煌国家级文化产业示范园区、酒泉故事文化产业园等文化创意产业园区建设，重点打造敦煌阳关等文化旅游小镇，启动《石窟中国》和《到全世界找敦煌》创作拍摄工作，支持发展会展、演艺和文化产品交易，不断提高文化软实力。

联动发展全域旅游。按照“全区域谋划、全产业联动、全要素配套、全时空统筹、全社会参与”的理念，大力推进西四县旅游一体化发展、东三县旅游一体化打造，抓好敦煌国家级和玉门、金塔省级全域旅游示范区创建。完成敦煌省级大景区建设任务，提升旅游景区基础设施水平，积极创建一批4A级景区，推进酒泉航天太空城等重点项目。加大休闲旅游、乡村旅游、健身旅游宣传推介力度，实施乡村旅游服务设施提升工程。深入开展全域旅游无垃圾、全线景观廊道基础改造提升、全业旅游服务质量提升、旅游厕所建设“新三年计划”等专项行动，打造精品丝路、航天科技、民族风情等精品旅游线路，力争旅游接待人数、旅游收入增加值增长20%以上。

培育发展现代服务业。深入推进“互联网＋流通”行动计划，构建电子商务新优势，实施县级中心带动乡村电商网点、信息平台带动生产要素整合、名优品牌带动优势产业扩张三大战略，实现网络服务一体化、要素互通便利化、产品销售高端化，电子商务交易额增长30%。依托铁路、公路、航空、口岸、煤电油运综合通道优势和通关一体化条件，打造“一带一路”国际物流节点城市。扶持发展家政服务、养老托幼、医疗保健等与多样化需求相适应的生活性服务业，培育壮大健康、养老等“幸福产业”。

积极发展外向型经济。充分利用“中新南向通道”，借助敦煌国际空港，加强与沿线省区市的经贸协作，推动形成陆海内外联动的开放格局。争取建设国家级制种出口基地，扩大省级外贸转型基地规模，培育出口龙头企业，提高制种、脱水菜、番茄酱等农产品出口档次，促进新能源装备、种子机械等工业品出口。积极与相关国家开展食品、农产品认证互认合作，提高食品农产品贸易水平。加强国际产能合作，探索与丝绸之路沿线国家合作发展的路径，支持大禹节水在南非、东盟兴建节水灌溉设备制造基地，鼓励农产品流通企业在哈萨克斯坦等国兴建农产品仓储物流项目，拓展酒泉农产品直销东盟通道。

5.建管并重提升服务，打造丝路魅力城市

牢固树立以人为核心的城市化发展理念，优化城市布局，完善城市功能，彰显城市特色，增强承载辐射能力，加快省域副中心城市建设步伐。

高起点完善城市规划。按照省域副中心城市定位，严格执行城市总体规划，加快编制酒泉城北片区、酒航路两侧等控制性详规和“智慧城市”“海绵城市”、综合交通、酒泉城区地下空间等专项规划。全力推进“多规融合”“城市双修”工作，完成瓜州、肃北、阿克塞“多规合一”规划，加快肃州、敦煌、玉门生态修复和城市修补规划编制。加强城市规划管理，深入推进违法建设专项治理五年行动计划，维护规划的严肃性和权威性。

高标准推进城市建设。加快酒泉新城区二期和高铁片区开发，加大城市道路、供热供气、垃圾处理、停车场等城市基础设施配套完善和改造维护力度。推进住房制度改革，加快棚户区、城中村和老旧楼院小区改造，改善群众居住条件。加强市政公用设施建设，实施敦煌市第三热源厂等清洁能源供暖项目，抓好城市生活垃圾分类制度改革试点，启动建设污水厂再生水利用和污泥处置设施。多点布局园林绿地和休闲广场，建设酒泉城区美溪公园，提升改造春光公园，建成区绿化覆盖率提高到38%以上。推进公共资源均衡配置，增强教育、医疗、文化、商贸等服务功能，努力让市民生活得更方便、更舒心、更美好。

高水平加强城市管理。盘活土地、空间、设施等城市资源，用好供水、供气、供热等有形资产，支持社会资本参与建设综合管廊等市政设施。加快“智慧城市”建设，完善“数字酒泉”地理信息系统，推进供热、供水、供气、排污等公用事业智能化管理，建设集运营管理、应急指挥、综合治理于一体的城市管理服务平台，建立综合型城市管理数据库，提高数字化城市管理运行水平。加快城市执法体制改革，理顺城市管理体制，完善城市网格化、精细化治理机制，全面落实“街长制”，解决交通拥堵、占道经营和脏乱差等问题，下大力气改善城市环境。

6.着力扩大有效投资，增强转型发展后劲

坚持把扩大有效投资作为稳增长、调结构、惠民生的有力抓手，加大项目谋划和资金争取，坚持招商引资借助外力，力促投资增速保持在合理区间。

多谋民生项目补短板。积极谋划一批农村道路、住房改造、扶贫攻坚、社会事业等重大项目，争取实施一批补齐发展短板、增加公共服务供给的民生项目。重点推进酒嘉绕城高速、G312酒泉过境段改造、肃州城市地下管廊、39个建制镇生活污水垃圾无害化处理等项目前期工作，开工建设柳敦客专、酒额铁路和肃航路至核产业园公路，加快敦当高速、柳敦高速、沙肃一级、敦煌方山口至七里镇公路建设进度，确保敦格铁路、肃航公路、马鬃山口岸公路建成通车。

聚焦生态项目强弱项。实施一批生态系统保护和修复重大工程，持续抓好退牧还草、草原治理、湿地保护与恢复项目。推进疏勒河、党河等中小河流治理，争取引哈济党工程取得突破，做好敦煌生态治理、河西走廊高效节水示范区项目前期工作，加快肃北马鬃山供水工程等续建项目进度。继续实施三北防护林、防沙治沙、城市防风林等生态建设工程，新建改造农田防护林400公里，绿色通道200公里，完成人工造林12万亩，风沙口治理6.5万亩，争取启动实施国土绿化行动。

狠抓产业项目增后劲。围绕国家产业布局调整和投资政策导向，谋划实施一批有机农业、节能环保、清洁生产、文化旅游、先进制造等方面的产业转型升级项目。全力推进瓜州常乐电厂、55万千瓦光热、水峡口水电站、金泉330千伏输电等能源项目，做好第二批光热项目争取工作，加快金塔金源等煤化工项目建设进度，启动实施三新硅业二期扩建及余热发电项目，做好肃州区垃圾发电项目前期工作，完成昌马抽水蓄能电站可行性论证，加大东方铝业硬质铝合金、白石头沟年产3000吨石墨精粉等非能源工业项目推进力度。

力促招商项目扩规模。认真落实省政府鼓励招商引资“24条”，制定出台促进招商引资激励政策，营造亲商、爱商、富商的良好发展环境，完善驻点招商、节会招商、委托招商、以商招商、小分队招商工作措施，大力开展全产业链招商，精准对接引进一批“高、新、强”项目。加强部门联动，靠实招商责任，强化代办制服务，把好投资强度、环境安全等准入关口，提高引进项目质量，促进招商引资上水平、见实效。积极推进政府与社会资本合作(PPP)，鼓励民营资本参与道路交通、市政设施、生态环保等特许经营项目和医疗、养老、教育等民生项目，力促民间投资回稳向好。

7.加大生态环境保护，加快生态文明建设

牢固树立绿水青山就是金山银山的理念，坚持“南护水源、中建绿洲、北拒风沙”，加强生态环境保护治理，促进生产空间集约高效、生活空间宜居适度、生态空间山清水秀，加快建设美丽酒泉。

大力实施重大生态治理工程。正确处理生态保护与经济社会发展的关系，扎实做好祁连山国家公园体制试点工作。加强自然保护区和林地林木管理，加快农田防护林体系建设，落实禁牧休牧和草畜平衡制度，依法依规做好矿山恢复治理，促进生态保护区补偿机制落地。坚持重大生态工程与区域、流域生态综合治理相结合，强化“三条红线”控制，抓好地下水超采区治理修复，深入实施疏勒河水流产权确权试点和敦煌全国水生态文明城市建设试点。

打好污染防治攻坚战。认真落实全域无垃圾三年专项治理方案和城乡环境综合整治实施意见，加快村容村貌整治、饮水安全提升、污水垃圾处理系统建设，完善城镇污水处理设施和排水管网，加快中水回用项目建设。深入实施大气、水、土壤污染防治行动计划，持续推进农业面源污染治理，继续实施化肥、农药零增长行动，加强工业排污、涉水企业、污水处理等重点行业污染物排放管控。开展工业集中区循环化改造，抓好清洁能源供暖示范工程和敦煌国家级新能源示范城市建设，加快工业锅炉改造、余热余压利用等节能项目建设，力促生态环境质量持续改善。

认真抓好生态环境问题整改。实行最严格的生态环境保护制度，汲取祁连山生态环境破坏问题深刻教训，落实生态文明建设主体责任，全面彻底整改中央和省环保督察反馈问题，确保违建违规项目和生产经营活动依法依规关停退出。严格主体功能区产业准入，科学划定基本农田保护区、重点生态功能区、生态环境敏感区和脆弱区管控边界，禁止一切不符合主体功能区、自然保护区功能定位的开发建设活动，把我们赖以生存的自然环境保护好。

加快推进生态文明体制改革。建立自然资源资产产权和有偿出让制度，严守生态保护红线和永久基本农田、城镇开发边界控制线，完善自然生态空间用途管制体系。落实最严格的耕地和水资源管理制度，深入推进“河长制”“湖长制”，着力构建覆盖全面、科学规范、管理严格的资源总量管理和节约利用制度框架。探索编制自然资源资产负债表，加强信息基础、监测预警和决策支持。推进生态文明建设目标评价考核，加大领导干部自然资源资产离任审计，形成推进生态文明建设的常态化机制。

8.持续保障改善民生，不断增进人民福祉

牢固树立以人民为中心的发展思想，持续加大民生保障和改善力度，提高公共服务共建能力、共享水平和均等化程度。

统筹推进社会事业。继续实施“全面改薄”工程，加大普惠性幼儿园奖补力度，下功夫解决城市学校大班额、农村学校师资薄弱、学生课业负担重的问题，提升普通高中办学水平，促进职业教育产教融合，努力让每个孩子都能享有公平而有质量的教育。认真落实健康酒泉战略，继续深化医疗改革，加快建设医联体和医养结合示范点，严格控制公立医院医疗费用，支持中医药事业发展，鼓励社会力量办医和外聘专家团队来酒诊疗。加强重大传染病控制工作，严防人间鼠疫、禽流感等疫情发生。促进生育政策落实，优化人口管理服务。完善“三馆一站”文化基础设施，投入运营市体育馆，提升文化惠民服务质量和水平。

强化社会保障工作。深入实施创业促进就业计划，加强劳务技能培训，强化“双创”扶持措施，城镇登记失业率控制在3.5%以内，高校毕业生就业率保持在85%以上。全面实施全民参保计划，完善异地就医直接结算和城乡居民医疗保险统一经办制度，深化机关事业单位养老保险制度改革，做好社保提标扩面、城乡居民低保、大病保险、困难群体救助和残疾人康复服务工作。构建和谐稳定劳动关系，落实农民工工资支付保障制度，开展治欠保支专项检查。启动实施新的三年棚改攻坚计划，健全多主体供给、多渠道保障、租购并举的住房保障制度。

着力办好惠民实事。从人民最关心最直接最现实的利益问题着手，重点抓好十件惠民实事:一是酒泉第七中学如期招生，新增招生规模3500个，启动第八中学及附属幼儿园前期工作。二是改造棚户区住房7728套。三是新建改造农村公路600公里、城市道路45公里。四是加快天然气入户工程，酒泉城区新增燃气用户3万户，基本实现全覆盖。五是建立经济困难老人养老服务补贴政策，为60岁及以上经济困难老人每月发放补贴100元。六是加快推进厕所革命，新建改建公共场所和旅游景区高标准厕所100座。七是建设市妇幼保健服务中心和市二院综合大楼。八是启动建设酒泉市老年大学。九是建设酒泉城区饮用水水质在线监测系统，让市民用上更加清洁安全的自来水。十是建设50个网货品牌站点，加盟特色专业户10000户，网销地方特色农产品10亿元。

9.切实加强社会治理，维护社会和谐稳定

完善社会治理体制，创新社会治理方式，着力打造共建共治共享的社会治理格局，推进社会治理能力现代化。

深入推进依法治市。广泛开展法治宣传教育，全面落实“七五”普法规划，增强全民法治意识。积极推动城乡建设管理、环境保护、历史文化保护等方面的立法工作。加快城市综合执法改革，建立统一的行政执法公众监督平台和跨部门行政执法信息平台。加大政府规范性文件监管力度，推行动态化、信息化管理。继续完善相对集中行政复议职权制度，提高行政复议办案质量。拓展法律援助领域，保障社会弱势群体的合法权益。加强法律服务队伍建设，落实法律顾问和公职律师、公司律师制度。

创新完善基层治理。建立完善以基层组织为核心、村民自治为基础的村民共建共治体系，促进形成村民自我管理、自我教育、自我约束的基层治理格局。加强和完善城乡社区治理，加快撤乡改镇步伐，做好村务公开标准化规范化试点工作，健全城乡社区服务功能和管理功能，开展农村社区建设示范创建活动，力争农村社区建设覆盖率达到95%以上。加快推行村和社区法律顾问工作，积极教育和引导群众在法律框架下解决问题，建设平安乡村、平安庭院，确保乡村社会充满活力、和谐有序。

促进民族团结宗教和顺。认真贯彻落实习近平新时代民族工作思想，牢牢把握“两个共同”的民族工作主题，更加关注支持民族县、民族乡发展，扎实开展全省第十五个民族团结进步宣传月活动，深入实施助推民族乡全面建成小康社会行动，积极争创全国民族团结进步示范市。加强民族地区文化交流，举办全市第八届少数民族传统体育运动会。提高宗教工作法制化水平，开展和谐寺观教堂创建活动，积极引导宗教与社会主义社会相适应，巩固宗教和顺、社会和谐的良好局面。

持续推进平安酒泉建设。完善安全生产责任体系，加大安全生产监管执法力度，狠抓安全生产源头防控，坚决防止和遏制各类安全生产事故发生。实施食品安全战略，健全食品药品监管体系，严守从农田到餐桌的每一道防线，确保公众饮食用药安全。着力化解信访积案和矛盾纠纷，防范和打击电信网络诈骗、非法集资、买卖个人信息等各类违法犯罪活动，启动建设“雪亮”工程，构建立体化、信息化社会治安防控体系，提升防灾减灾救灾能力，切实保障人民群众生命财产安全。

全面加强政府自身建设

新时代开启新征程，新时代要有新作为。各级政府要高举中国特色社会主义伟大旗帜，全面贯彻党的十九大精神和中央、省市各项决策部署，不断提高执政为民能力，努力建设人民满意政府。

一要旗帜鲜明讲政治。始终把对党绝对忠诚作为干事创业、做好工作的思想基础，严守政治纪律和政治规矩，牢固树立政治意识、大局意识、核心意识、看齐意识，坚决维护以习近平同志为核心的党中央权威和集中统一领导，自觉在思想上政治上行动上同党中央保持高度一致。坚决贯彻中央和省市委决策部署，有力推动稳增长、促改革、调结构、惠民生、防风险各项任务落实。注重从政治上观大局、看问题，增强政治警觉性，提高政治鉴别力，自觉把讲政治贯穿于工作落实全过程，落实到改革发展稳定各项工作中，落实到打好三大攻坚战的具体实践中，确保政府工作始终沿着正确方向扎实推进。

二要增强本领提效能。深入领会习近平新时代中国特色社会主义思想，把学习党的十九大精神作为政治必修课，在学懂弄通做实上下功夫，对照增强“八个本领”的要求，扎实开展“不忘初心、牢记使命”主题教育，深入推进“两学一做”学习教育常态化制度化，努力提高政治素养和思想理论水平。准确理解把握新时代社会主要矛盾的变化，学习专业知识，提升业务素质，化解能力危机，不断适应新时代新形势新要求，着力提高行政效能和办事效率。

三要依法行政促公开。扎实推进法治政府建设，加强法律法规学习，提高依法行政水平，按照法定权限履行职责、行使权力，运用法治思维解决问题、化解矛盾，依据法定程序科学民主决策、推动改革发展。自觉接受人大依法监督、政协民主监督，接受司法、舆论和社会监督，认真办理人大代表建议和政协委员提案。健全依法行政制度体系，严格落实行政决策程序，完善重大决策风险评估机制。深入推进严格规范公正文明执法，强化行政执法监督，建立权责统一、权威高效的依法行政体制。加大政务公开力度，扩大信息公开范围，完善“三张清单一张网”，提高政府工作透明度，让权力在阳光下运行。

四要改进作风抓落实。人民对美好生活的向往就是我们的奋斗目标。牢记全心全意为人民服务的根本宗旨，紧扣贯彻落实十九大精神，紧扣经济社会发展实际，紧扣人民群众生产生活，大兴调查研究之风，多谋为民利民之策，在防范化解重大风险、精准脱贫、污染防治、乡村振兴、新旧动能转换等重点工作落实上，拿出切实可行的工作建议和措施办法。坚持“实”字当头、“干”字为先，增强创新意识、实干意识和服务意识，争做实干家，不做清谈客，真心实意为企业和群众排忧解难。落实激励机制和容错纠错机制，为敢于担当、踏实做事、不谋私利的干部撑腰鼓劲，营造想干事、能干事、干成事、不出事的良好氛围。深入推进“三纠三促”专项行动，坚决整治不担当、不作为、弄虚作假等行为，促进各项决策部署抓实见效。

五要廉洁从政树形象。认真落实党风廉政建设主体责任，严格执行中央“八项规定”精神和省市有关要求。坚决全面彻底肃清王三运、马光明流毒和影响，严肃查处发生在群众身边的不正之风和腐败问题，着力解决隐形变异“四风”问题，用铁的纪律加强和规范政府各项工作。加强预算约束和财政管理，严管公共资金资产资源，严控“三公”经费，坚持勤俭办一切事业。积极支持监察体制改革，加强行政监察和审计监督，健全权力运行制约和监督机制，加大对工程建设、政府采购、土地出让等重点领域的监管力度，从源头上遏制和预防腐败行为，努力营造风清气正的良好政治生态。

各位代表，新时代赋予新使命，新目标开启新征程。让我们紧密地团结在以习近平同志为核心的党中央周围，在省委、省政府和市委的坚强领导下，凝聚全市各族人民的智慧和力量，不忘初心，牢记使命，勇于担当，真抓实干，为加快活力小康生态魅力幸福酒泉建设而努力奋斗!
